# Association between systemic inflammatory markers and otosclerosis: a retrospective case–control study

**DOI:** 10.3389/fneur.2026.1794083

**Published:** 2026-04-07

**Authors:** Hüseyin Işık, Deniz Baklaci

**Affiliations:** Faculty of Medicine, Zonguldak Bulent Ecevit University, Zonguldak, Türkiye

**Keywords:** hearing-disorders, inflammation, neutrophil-to-lymphocyte ratio, otosclerosis, systemic immune-inflammation index

## Abstract

**Objective:**

Otosclerosis is a localized metabolic bone disease of the otic capsule with an inflammatory component. This study investigated whether otosclerosis is associated with alterations in systemic inflammatory indices, namely the Neutrophil-to-Lymphocyte Ratio (NLR) and the Systemic Immune-Inflammation Index (SII).

**Materials and methods:**

This retrospective case–control study included 52 patients with otosclerosis who underwent stapedotomy and 50 control subjects who underwent septoplasty for isolated nasal septal deviation between January 1, 2023 and January 1, 2024. Preoperative venous blood samples were obtained within 7 days before surgery, between 08:00 and 10:00 a.m. after overnight fasting. Complete blood counts were analyzed using an automated hematology analyzer (UniCel DxH 800, Beckman Coulter, USA). SII was calculated as (platelet × neutrophil)/lymphocyte, and NLR as neutrophil/lymphocyte. Group comparisons, multivariable logistic regression (adjusted for age and sex), and ROC curve analyses were performed.

**Results:**

SII was significantly higher in the otosclerosis group than in controls (599.1 ± 154.3 vs. 503.5 ± 114.8; *p* = 0.041). NLR was numerically higher in otosclerosis (2.14 ± 0.8 vs. 1.89 ± 0.6) but did not reach statistical significance (*p* = 0.107). Individual components showed no significant between-group differences in neutrophil count (4.96 ± 1.53 vs. 4.73 ± 1.99 × 10^3^/μL; *p* = 0.516) or platelet count (279.22 ± 55.71 vs. 266.63 ± 74.98 × 10^3^/μL; *p* = 0.337), while lymphocyte count was significantly lower in otosclerosis (2.31 ± 0.65 vs. 2.50 ± 0.84 × 10^3^/μL; *p* = 0.019). In multivariable analysis, SII (per 100-unit increase) remained independently associated with otosclerosis (OR = 1.24, 95% CI: 1.00–1.53; *p* = 0.047). ROC analysis demonstrated moderate discrimination for SII (AUC = 0.658, 95% CI: 0.501–0.815).

**Conclusion:**

SII was independently associated with otosclerosis, suggesting a subtle systemic immune–inflammatory signature. Given the borderline significance and moderate ROC performance, these findings should be interpreted as exploratory and require confirmation in larger prospective cohorts.

## Introduction

Otosclerosis is a localized metabolic bone disease with a significant inflammatory component (1). Although its etiopathogenesis is multifactorial, a growing body of evidence points to a significant inflammatory component in its pathogenesis. This is supported by the findings of viral involvement, specifically the persistence of measles virus RNA in active otosclerotic foci, which triggers a local inflammatory cascade (1). Furthermore, this local inflammation is characterized by the upregulation of pro-inflammatory cytokines, such as Tumor Necrosis Factor-alpha (TNF-*α*), which plays a crucial role in bone resorption and new bone formation cycles typical of the disease (2).

Systemic inflammatory markers, such as the neutrophil-to-lymphocyte ratio (NLR) and systemic immune-inflammation index (SII), are established and cost-effective indicators of an individual’s immune status, with prognostic value in various diseases (3–7). Although otosclerosis is traditionally viewed as a localized bone disorder, emerging evidence suggests a localized cytokine-driven inflammatory process. However, it remains unclear whether localized inflammation has measurable systemic components. Therefore, this study aimed to challenge this traditional view by investigating the relationship between otosclerosis and systemic inflammatory markers. We hypothesized that patients with otosclerosis would exhibit elevated NLR and SII compared to a healthy control group, suggesting that accessible biomarkers could have potential utility in assessing the systemic inflammatory nature of the disease.

## Materials and methods

This retrospective case–control study included 52 patients diagnosed with otosclerosis who underwent stapedotomy and 50 control subjects who underwent septoplasty for isolated nasal septal deviation at the Ear, Nose, and Throat Clinic of Zonguldak Bülent Ecevit University Faculty of Medicine Hospital between January 1, 2023 and January 1, 2024.

Otosclerosis was diagnosed based on progressive conductive hearing loss (air-bone gap ≥20 dB on pure-tone audiometry), type A/As tympanogram, absent stapedial reflexes, and intraoperative confirmation of stapes fixation. The control group comprised patients with isolated nasal septal deviation, a mechanical condition with no known systemic inflammatory component. Septoplasty patients were selected as the control group because nasal septal deviation is a localized mechanical condition with no established systemic inflammatory component. These patients typically represent otherwise healthy individuals undergoing elective surgery, allowing for a comparable perioperative laboratory assessment environment. To minimize selection bias, we ensured that no control subject had active infection, chronic inflammatory disease, or any systemic disorder known to affect hematological parameters.

The inclusion criteria were age 18–65 years and the absence of systemic diseases associated with chronic inflammation. Exclusion criteria included: BMI ≥ 30 kg/m^2^, current smoking or cessation within 6 months, autoimmune diseases, diabetes mellitus, active malignancies, chronic infections, use of systemic corticosteroids, NSAIDs, statins, or immunosuppressants within 3 months, and any acute infection or fever within 2 weeks of blood sampling. Preoperative venous blood samples were obtained within 7 days before surgery, between 08:00 and 10:00 a.m., following an overnight fast. Complete blood counts were analyzed using an automated hematology analyzer (UniCel DxH 800, BECKMAN COULTER, USA). The Systemic Immune-Inflammation Index (SII) was calculated as follows: SII = (platelet × neutrophil)/lymphocyte, and NLR as follows: NLR = neutrophil/lymphocyte.

The study protocol was approved by the Ethics Committee of Zonguldak Bülent Ecevit University (Date: February 7, 2024; No: 2024/02). For this retrospective study, written informed consent was obtained from all patients whose data were accessible, and the study was conducted in strict accordance with the principles of the Declaration of Helsinki.

### Statistical analysis

Statistical analyses were performed using SPSS version 26.0 (IBM Corp., Armonk, NY, USA) and Python-based statistical libraries (SciPy and Statsmodels). The normality of the data distribution was assessed using the Shapiro–Wilk test.

Descriptive statistics are presented as mean ± standard deviation (SD) for continuous variables and as frequencies and percentages for categorical variables. For the comparison of groups, Welch’s t-test was used for continuous variables (SII, NLR, PLT, and age), and the chi-square test was used for categorical variables (sex).

Multivariable logistic regression models were constructed to identify independent predictors of otosclerosis. To avoid multicollinearity between highly correlated markers, the NLR and SII were analyzed in two separate models, both adjusted for age and sex. The SII values were scaled by 100 units to provide more interpretable odds ratios (OR). The diagnostic performance of the markers was evaluated using Receiver Operating Characteristic (ROC) curve analysis, and the Area Under the Curve (AUC) with 95% confidence intervals (CI) was calculated. Optimal cutoff values were determined using the Youden Index. Statistical significance was set at *p* < 0.05.

## Results

### Demographic and clinical characteristics

A total of 102 participants (52 patients with otosclerosis and 50 healthy controls) were included in the final analysis. The mean age was 35.17 ± 10.1 years in the otosclerosis group and 36.29 ± 10.3 years in the control group, with no statistically significant difference between the groups (*p* = 0.707). Gender distribution was also similar, with 11 males (21.1%) in the otosclerosis group and 13 males (26.0%) in the control group (*p* = 0.564).

### Comparison of hematological markers

A comparison of the systemic inflammatory markers between the two groups is presented in Table 1. The Systemic Immune-Inflammation Index (SII) was higher in the otosclerosis group (599.1 ± 154.3) than in the control group (503.5 ± 114.8), reaching statistical significance (*p* = 0.041). The Neutrophil-to-Lymphocyte Ratio (NLR) was also higher in patients with otosclerosis (2.14 ± 0.8) than in the control group (1.89 ± 0.6), although this difference did not reach statistical significance in the cohort (*p* = 0.107). Analysis of individual hematological components revealed that the mean neutrophil count was 4.96 ± 1.53 × 10^3^/μL in the otosclerosis group and 4.73 ± 1.99 × 10^3^/μL in the control group (*p* = 0.516). The mean platelet count was 279.22 ± 55.71 × 10^3^/μL and, 266.63 ± 74.98 × 10^3^/μL, respectively, (*p* = 0.337). The mean lymphocyte count was significantly higher in the control group (2.50 ± 0.84 × 10^3^/μL) compared to the otosclerosis group (2.31 ± 0.65 × 10^3^/μL) (*p* = 0.019).

**Table 1:** Comparison of demographic characteristics and hematological parameters between patients with otosclerosis and healthy controls.

Parameter	Group	*n*	Mean	SD	*p*-value
SII	Control	50	503.5	114.8	**0.041**
	Otosclerosis	52	599.1	154.3	
NLR	Control	50	1.89	0.6	0.107
	Otosclerosis	52	2.14	0.8	
Neu (10^3^/μL)	Control	50	4.73	1.99	0.516
	Otosclerosis	52	4.96	1.53	
Lym (10^3^/μL)	Control	50	2.50	0.84	**0.019**
	Otosclerosis	52	2.31	0.65	
Plt (10^3^/μL)	Control	50	266.63	74.98	0.337
	Otosclerosis	52	279.22	55.71	

*p* < 0.05. SII, systemic immune-inflammation index; NLR, neutrophil-to-lymphocyte ratio; SD, standard deviation; Neu, neutrophil; Lym, lymhocyte; Plt, platelet.

Bold values indicate statistically significant independent predictors (*p* < 0.05).

### Multivariable logistic regression analysis

Two separate multivariable logistic regression models were constructed to determine the independent predictors of otosclerosis, adjusting for age and sex (Table 2). In Model 1, SII (analyzed per 100-unit increase) was identified as a significant independent predictor of otosclerosis (OR = 1.24, 95% CI: 1.00–1.53, *p* = 0.047). In Model 2, NLR showed a positive association with otosclerosis (OR = 1.83, 95% CI: 0.93–3.59), but the association was not statistically significant (*p* = 0.081). In both models, age and sex were not significant predictors of the disease (*p* > 0.05), confirming the success of the matching.

**Table 2:** Multivariable logistic regression analysis identifying independent predictors of otosclerosis.

Model	Variable	OR	95% CI	*p*-value
Model 1	SII (per 100 units)	1.24	1.00–1.53	**0.047**
	Age	0.99	0.93–1.05	0.786
	Sex (male)	0.47	0.14–1.63	0.236
Model 2	NLR	1.83	0.93–3.59	0.081
	Age	0.98	0.93–1.05	0.614
	Sex (male)	0.44	0.12–1.55	0.202

OR, odds ratio; CI, confidence interval; *p* < 0.05.

Model 1: SII/100, age, sex; Model 2: NLR, age, sex.

Bold values indicate statistically significant independent predictors (*p* < 0.05).

### ROC curve analysis

**Figure 1:**
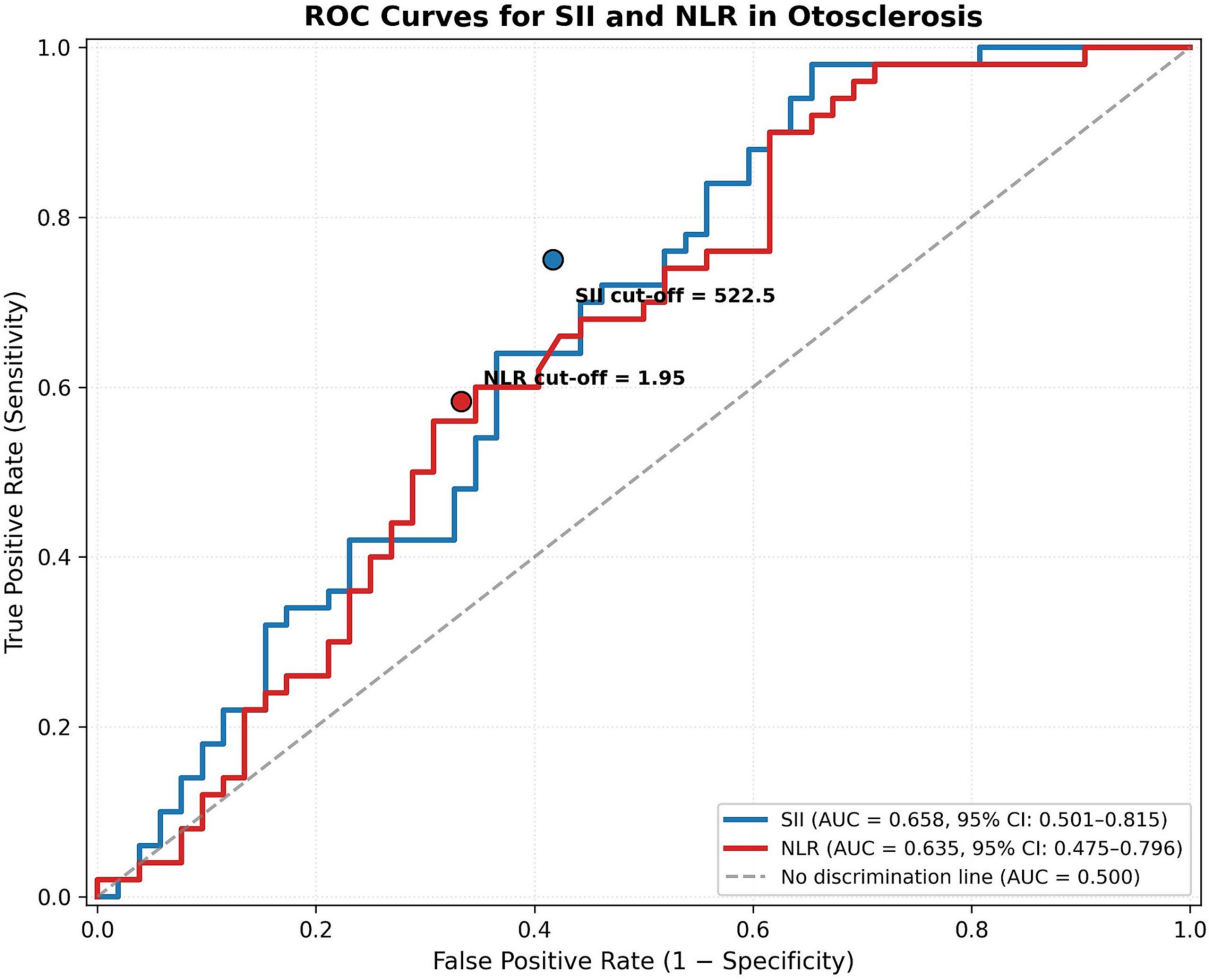
Receiver operating characteristic (ROC) curves of systemic immune-inflammation index (SII) and neutrophil-to-lymphocyte ratio (NLR) for the prediction of otosclerosis.

ROC curve analysis was performed to evaluate the diagnostic performance of SII and NLR in the cohort (Table 3). The Area Under the Curve (AUC) for SII was 0.658 (95% CI: 0.501–0.815). Using a cutoff value of 522.5 for SII, the sensitivity and specificity for predicting otosclerosis were 75.0 and 58.3%, respectively. For NLR, the AUC was 0.635 (95% CI: 0.475–0.796), with a sensitivity of 58.3% and specificity of 66.7% at a cutoff value of 1.95 (Figure 1).

**Table 3:** Diagnostic performance of SII and NLR in discriminating patients with otosclerosis from healthy controls.

Marker	AUC	95% CI	Cut-off	Sensitivity	Specificity
NLR	0.635	0.475–0.796	1.95	58.3%	66.7%
SII	0.658	0.501–0.815	522.5	75.0%	58.3%

95% CI estimated using bootstrap (5,000 resamples).

## Discussion

The present study investigated the association between systemic inflammatory markers and otosclerosis using a case–control design. The principal finding of this analysis was that the Systemic Immune-Inflammation Index (SII) remained an independent predictor of otosclerosis after controlling for age and sex, whereas the Neutrophil-to-Lymphocyte Ratio (NLR) demonstrated only a non-significant trend. It is crucial to emphasize that the observed elevation in SII levels represents a relative, subclinical increase rather than overt systemic inflammation. Furthermore, our findings indicate a statistical association rather than a causal link between systemic immune activation and the pathogenesis of otosclerosis. An important aspect of the present results is that the absolute SII and NLR values observed in both the otosclerosis and control groups were within the reference ranges reported in large population-based studies (8, 9). This observation does not undermine the relevance of the findings of the present study. Population reference values are designed to detect overt or clinically apparent inflammation across heterogeneous cohorts, whereas the aim of the present study was to identify subtle disease-specific differences between groups. Accordingly, our results should not be interpreted as evidence of clinically abnormal systemic inflammation in otosclerosis but rather as an indication of a relative elevation of low-grade subclinical inflammation compared with healthy individuals. This relative elevation suggests that even if the markers remain within ‘normal’ laboratory limits, they may reflect a **s**ubclinical systemic response to localized metabolic activity within the otic capsule. Such subtle systemic changes may be pathophysiologically relevant, particularly in chronic diseases characterized by localized and persistent inflammatory activity.

Otosclerosis is a complex disorder characterized by abnormal bone remodeling within the otic capsule, ultimately resulting in progressive conductive hearing loss (10). In patients with otosclerosis, abnormal bone deposits encase and attach to the ossicles, disrupting the mechanical transmission of sound and resulting in conductive hearing loss. Although its precise etiology remains incompletely understood, a growing body of evidence supports the involvement of inflammatory and immune-mediated mechanisms in its pathogenesis. Prior experimental studies have demonstrated increased production of pro-inflammatory cytokines, such as interleukin1β and interferon-inducible protein10, in otosclerotic bone cell cultures, suggesting an intrinsically activated inflammatory environment within the affected tissue (11). Liktor et al. suggested that pharmacological treatments, including steroids, nonsteroidal anti-inflammatory drugs, and vitamin D, might be effective during the early inflammatory phase of otosclerosis (12).

Routine blood tests for evaluating inflammatory processes are often useful for the early diagnosis of various diseases (13). Systemic inflammatory markers, such as neutrophil, platelet, and lymphocyte counts, NLR, and SII, which have become prominent in recent years, provide crucial information about an individual’s immune status. The prognostic importance of NLR has been demonstrated in many diseases with an inflammatory etiology. Brescia et al. found that preoperative NLR, eosinophil-to-lymphocyte ratio, and basophil-to-lymphocyte ratio were significantly higher in patients with recurrent chronic rhinosinusitis with nasal polyps than in those without (14). In contrast, Arli et al. found no significant difference in NLR between patients with otosclerosis and controls in their study of 30 patients (15). Consistent with their findings, our analysis revealed that although NLR levels were higher in patients with otosclerosis, the difference was not statistically significant (*p* = 0.107).

The Systemic Immune-Inflammation Index (SII) is another systemic inflammatory marker that has been frequently studied in recent years. Calculated from three key inflammatory cell types (neutrophils, platelets, and lymphocytes), the SII is thought to provide a more comprehensive representation of the host immune and inflammatory balance (9, 16). Consequently, similar to NLR, its efficacy as a useful indicator for predicting clinical outcomes has been demonstrated in many inflammatory diseases, particularly cancer, thus garnering significant interest from researchers (17, 18). In the field of otology, SII has recently been evaluated in inflammatory middle-ear diseases, where it was found to differ significantly between mucosal and squamosal chronic otitis media phenotypes, suggesting that systemic indices can reflect specific temporal-bone inflammatory endotypes (19). Furthermore, the prognostic value of SII has been demonstrated in otologic surgical contexts; for instance, higher preoperative SII levels were associated with graft failure in patients undergoing tympanoplasty, highlighting its utility as a pragmatic adjunct marker in ENT practice (20). Ulu et al. suggested that the SII can predict the prognosis of sudden idiopathic hearing loss (21). Ekici et al. examined the relationship between NLR and SII in the differential diagnosis of benign and malignant masses in 134 patients with parotid gland tumors who underwent parotidectomy. They found that the mean NLR was significantly higher in malignant masses, whereas there was no significant difference in the SII values (22). In a study of 514 patients with TI-RADS grade 3 thyroid nodules, Deng et al. reported that the leukocyte, neutrophil, and platelet counts, NLR, PLR, and SII values were significantly higher in the malignant nodule group than in the benign nodule group (6). Kınar et al. reported that neutrophil count, NLR, and SII values were higher in patients with Bell’s palsy than in controls, suggesting that SII could be a prognostic marker for this condition (23). Our results, which showed significantly higher SII values in patients with otosclerosis, suggest a potential systemic inflammatory milieu that may coexist with the localized inflammation within the otic capsule, rather than serving as a direct evidence of pathogenesis. In this context, the SII may offer theoretical advantages over the NLR as a marker of disease-related inflammation. By integrating neutrophil, platelet, and lymphocyte counts into a single index, the SII reflects not only innate immune activation but also platelet-mediated inflammatory and thrombo-inflammatory pathways, while accounting for adaptive immune regulation through lymphocyte levels. This composite structure may allow the SII to capture the immune–inflammatory balance more comprehensively than ratios based on two cell types alone. The persistence of a significant association between the SII and otosclerosis in the logistic regression model supports this notion and suggests that platelet-related inflammatory mechanisms might be associated with the disease’s systemic signature.

The diagnostic utility of these markers was further evaluated using ROC curve analyses. In our cohort, SII demonstrated moderate discriminative performance with an AUC of 0.658, which was superior to that of NLR (AUC = 0.635). At a determined cutoff value of 522.5, the SII exhibited a high sensitivity of 75.0%, suggesting its potential as a screening tool for identifying patients with a higher inflammatory burden. However, the relatively low specificity (58.3%) indicates that the SII should not be utilized as a standalone diagnostic test but rather as a supplementary marker alongside clinical and audiological findings to better understand the systemic inflammatory status of patients with otosclerosis. However, given the borderline statistical significance of the SII (*p* = 0.047), the relatively wide confidence interval of the odds ratio (95% CI: 1.00–1.53), and the moderate discriminative ability shown by the ROC analysis (AUC = 0.658), these findings should be interpreted with caution. Our results should be viewed as exploratory rather than definitive evidence of a diagnostic role for SII in otosclerosis, highlighting the need for larger, multi-center prospective studies to confirm these preliminary observations.

This study has several limitations. First, the retrospective, single-center nature of the study limits causal inference and increases the risk of selection bias; therefore, the observed associations should be interpreted as hypothesis-generating rather than definitive evidence of an inflammatory mechanism in otosclerosis. Second, although blood samples were collected under standardized institutional conditions (between 08:00–10:00 a.m., following an overnight fast, within 7 days before surgery), the retrospective nature of the study precludes full verification of pre-analytical compliance for all participants, which may represent a minor source of variability. Third, the selection of the control group may limit the study’s interpretation. The use of a surgical control group may have biased the inflammatory indices toward null, potentially underestimating the true differences between the groups. Fourth, this study focused on patients with established otosclerosis scheduled for stapedotomy, meaning that the results likely reflect a later-stage/chronic disease phenotype and may not be generalizable to early stage otosclerosis or to patients managed conservatively. Finally, we did not evaluate the correlations between inflammatory markers and disease severity or clinical phenotype (e.g., hearing thresholds, air–bone gap, symptom duration, and bilateral involvement), nor did we assess other inflammatory biomarkers (e.g., CRP) that could help contextualize SII/NLR. The modest discrimination observed in the ROC analysis indicates that the SII should not be considered a standalone diagnostic marker, and its potential clinical utility remains to be clarified.

Owing to the retrospective and cross-sectional nature of this study, we could not establish a causal relationship between systemic inflammation and the development of otosclerotic foci. Rather than serving as a diagnostic tool, the SII may provide insights into the systemic immune-inflammatory milieu accompanying otosclerosis. Because of the retrospective design, no *a priori* sample size or power calculation was performed. Consequently, the study may be underpowered to detect small-to-moderate between-group differences—particularly for NLR—and the possibility of type II error cannot be excluded. In addition, the modest sample size resulted in relatively wide confidence intervals for the AUC estimates and odds ratios, indicating limited statistical power and precision. As a result, the reported effect sizes and cut-off performance parameters may be unstable and require confirmation in larger prospective cohorts.

## Conclusion

In this case–control study, SII was independently associated with the presence of otosclerosis after adjustment for age and sex, whereas NLR demonstrated only a non-significant trend. These findings support the presence of a subtle systemic inflammatory signature in otosclerosis and suggest that SII may more robustly capture disease-associated immune–inflammatory alterations than NLR. Furthermore, ROC analysis confirmed that the SII provides a more reliable discriminative value than the NLR in this population, although its moderate specificity suggests that it is best used as a complementary rather than a primary diagnostic tool. However, given the retrospective design, reduced sample size, and potential residual confounding, the results should be interpreted with caution. Larger prospective studies incorporating standardized blood sampling and correlations with audiological severity are needed to validate the SII as a clinically meaningful biomarker and to clarify its role in the pathophysiology and risk stratification of otosclerosis. Future longitudinal studies are required to determine whether systemic inflammation precedes the onset of otosclerosis or occurs as a secondary response to localized bone remodeling. In conclusion, SII may more robustly capture subclinical immune-inflammatory alterations in otosclerosis than NLR. Future longitudinal studies are required to clarify the temporal relationship and determine whether this low-grade systemic signature has any predictive value for disease progression.

## Data Availability

The raw data supporting the conclusions of this article will be made available by the authors, without undue reservation.
